# Older Adults’ Reflections on Ageism and Racism

**DOI:** 10.1093/geroni/igaa057.091

**Published:** 2020-12-16

**Authors:** Janna Heyman, Colette Phipps, Peggy Kelly, Linda White-Ryan

**Affiliations:** 1 Fordham University, West Harrison, New York, United States; 2 Westchester County, West Harrison, New York, United States

## Abstract

The older population is becoming more racially and ethnically diverse. By the year 2050, 39% of those 65+ will be from minority groups, up from 21% in 2012 (Ortman et al., 2014). These figures have significant implications for aging policy, including concerns over ageism and racism. Discrimination can take many forms, and can be present in legislation, advertising, attitudes, the workplace, and the health care system (Snaedal, 2015). The present study examines perceptions of racial and age discrimination of older adults living in the community and its impact on their quality of life. Using a cross-sectional design, 134 participants over the age of 60 were surveyed at three senior centers with ethnically diverse populations. The Attitudes to Aging Questionnaire (AAQ-24) was used to assess participants’ perceptions and experiences with aging and perceptions of racism were assessed using an adaptation of the Modern Racism Scale. Findings from the AAQ-24 revealed an average score of 27.1 (SD=6.66) for psychosocial loss, 28.3 (SD=5.34) for physical change, and 30.5 (SD=4.65) for psychological growth, indicating moderately high levels of ageism. For the racism scale, the average total score for all respondents was 34.4 (SD=7.05), also moderate. This study helps shed some light on what older adults feel about the aging, as well as their concerns with racial discrimination. The insights gained from older adults’ experiences and perceptions can help shape policies for future generations.

